# Exploring the Linkages of Digital Food Communication and Analog Food Behavior: A Scoping Review

**DOI:** 10.3390/ijerph19158990

**Published:** 2022-07-24

**Authors:** Tina Bartelmeß, Jasmin Godemann

**Affiliations:** 1 Faculty of Life Sciences: Food, Nutrition and Health, Campus Kulmbach, University of Bayreuth, Fritz-Hornschuch-Strasse 13, 95326 Kulmbach, Germany; 2 Faculty of Agricultural Sciences, Nutritional Sciences and Environmental Management, Department of Consumer Research, Communication and Food Sociology, Justus-Liebig-University Giessen, Senckenbergstrasse 3, 35390 Giessen, Germany; jasmin.godemann@fb09.uni-giessen.de

**Keywords:** social media, food communication, food behavior, linkages, social-ecological model

## Abstract

The linkages of digital food communication on social media platforms and analog food behavior of social media users are widely discussed in media and research, but less differentiated. Due to the interdisciplinary nature of the research field, the scientific studies are characterized by great heterogeneity in approaching the role of communication and modelling of food behavior, and thus also the conclusions on how digital food communication might be linked to analog food behavior. There is still much uncertainty regarding the relationship and underlying assumptions between digital communication and analog action. The rationale of this scoping review is to systematically summarize the findings of this heterogeneous body of knowledge. The importance and originality of this review are that it focuses explicitly on studies that provide insights into the nexus of digital food communication and analog food behavior, be it in the theoretical foundation, the results, or their interpretation. It draws on a socio-ecological model of food behavior that depicts food behavior variables in different domains and uses a differentiated categorization of food behavior (food choice, dietary intake, and eating behavior) to synthesize the results. Using the Web of Science and PubMed databases, 267 abstracts were identified and screened, of which 20 articles met the inclusion criteria and were selected for full-text analysis. The review offers some important insights on how different variables of the socio-ecological model of food behavior are related to digital food communication and different areas of analog food behavior. This review provides a more discerning understanding of which aspects of analog food behavior may be linked to social media food communication and in which ways. Implications are derived to reflect the role of communication in previous models of food behavior by adding a more nuanced and cross-cutting understanding of food communication.

## 1. Introduction

The emergence of and pervasive practice of using social media platforms (e.g., Instagram, Facebook, and YouTube) have irrevocably changed the actors, modalities, public arenas, and inescapable presence of food communication in everyday life. By July 2021, 56.8 percent of the world’s population uses social media for an average of 2.5 h per day [1]. Especially among adolescents, the use of social media has long been a routine part of daily life [2] and the recent COVID-19 pandemic and related restrictions on analog social life have increased this usage further [3,4]. Each social media platform differs according to the demographics of its main users, its modalities, and its thematic foci—but the subject area of food is of high importance on almost all platforms and users engage with it in different platform-specific ways (textual, visual, audio-visual, likes, shares, retweets, etc.) [5,6,7].

Since the early 2000s, the emergence of social media platforms has structurally changed food communication, which can be summarized under the heading ‘demotic turn’ [8]. Before the digital turn, it was primarily professionalized food communication actors who were attributed interpretative power over food and nutrition issues. With social media platforms, today it is especially everyday actors who make food-related content and patterns of interpretation publicly accessible and available with their digital food communication and thus achieve a high level of attention [7,9,10]. Ordinary people become experts on ‘good’ nutrition and food-related choices by routinely revealing their experiences with food, expressing their opinions, and sharing them on these platforms. Once they reach a certain level of appeal, they can become so-called ‘food influencers’, depending on the number of people they reach and who follow them [11,12]. Consequently, traditional hierarchical instances of food communication are challenged and compete with new actors, and forms of public knowledge transfer that now define ‘good food’ [13]. Beyond traditional nutrition education or persuasive marketing communication, the communication of these new players in public food communication does not necessarily pursue the intention of influencing the recipients’ behavior. Notwithstanding, public media and recent academic studies, particularly on the food influencer phenomenon, have increasingly focused on potential influence, claiming that food communication on social media influences users’ analog food behavior and has implications for daily life [3,14,15].

Although the number of studies examining the use of social media and its influence on food and health behavior has increased significantly over the past decade [5,16,17], there has been no systematic analysis and modelling of *how* food communication on social media platforms affects users’ food behavior in their analog lives. By analog food behavior, this study refers to non-virtually performed food-related actions in people’s real lives. Existing studies each focus on different aspects, platforms, and social media communication modes. As for example in the most recent studies on the influence of visual communication on food behavior [18], analyzing message characteristics [19] and testing the effect of different nutrition-related messages via various social media channels on eating outcomes [20,21] or on the impact of social media (constructions) on pursuing specific diets in analog life [22,23,24]. However, a synthesis of previous findings has not been sufficiently documented. In addition, the research field is highly interdisciplinary due to the interfaces with behavioral, nutritional, media, cultural, and informatics research, and it is often unclear which understanding of communication and whether and which theories or models of food behavior underlie these studies and how comprehensive they are. As a result, the existing studies do not share a common object of knowledge as a reference basis. Conclusions regarding the actual linkages of digital food communication and analog food behavior can only be drawn to a limited extent from studies that are not based on a theory or model of food behavior, since it is not clear whether and which theory of food behavior or which variables of which behavior model were examined in the studies and how these variables are affected by communication and co-determine food behavior.

This review offers some important insights by providing a differentiated overview of how the impact of communication on behavior is approached in relevant studies. By systematically and critically reviewing previous findings on the linkages between digital food communication and analog food behavior, this review aims to contribute to a deeper understanding of what can ultimately be inferred about the impact of social media food communication on users’ food behavior in everyday life. Thus, the focus of the review is on how digital food communication affects analog food behaviors rather than the other way around, even though people interact with social media and there is no unidirectional influence. In its analytical approach, the review is original in that it provides a clarification of how communication and food behavior are defined and understood in current research, uncovering implicit assumptions. Understanding the implicit assumptions of previous findings on the linkages of digital food communication and analog food behavior in more detail offers an important opportunity to advance the consideration of communication and media in current theorizing and modelling of food behavior, and to expand previous approaches with a more contemporary perspective on modern social communication forms and modes. The review also provides avenues for actors in political food, health, and consumer communication, as the study highlights useful points to reflect on the potential success and targeting of their communication on social media platforms aimed at influencing users’ food behavior.

## 2. Analytical Frames: Food Behavior Areas and Social-Ecological Model of Food Behavior

Food behavior is often studied as food intake or healthiness of consumed foods [25]. Accordingly, several studies follow this approach when examining food behaviors relating to social media, operationalizing the influence as the consumption of nutrient-dense foods or the portion sizes chosen after exposure to social media content [18]. This review is based on a more complex understanding of food behavior. It aims to reveal how digital food communication affects people’s analog behavior by understanding how communication links and affects the various determinants and variables underlying food behavior, and in this way could influence individuals’ food-related choices and courses of action. Therefore, this review largely excludes experimental studies that focus exclusively on the effects of dietary intake following exposure to food-related content on social media as laboratory stimuli, with less focus on communication on social media platforms per se and intermediary perceptual processes. The review thus underscores the assumption of social-ecological modelling of food behavior [26] that there is no direct influence of communication on food behavior outcomes (e.g., food intake and portion sizes). Instead, determinants at various upstream levels are assumed to be modifiable by digital food communication processes and subsequently, are likely to influence analog food-related decisions and actions in combination with other determinants and conditions.

To distinguish virtual food-related media behaviors from everyday food behaviors, the review uses the terms ‘digital food communication’ and ‘analog food behaviors’. However, digital food communication practices are often directly linked to analog food behaviors (e.g., watching a recipe video on YouTube while cooking or eating in the cafeteria while posting a picture of the plate on Facebook) [27]. The term ‘analog food behavior’ is used here as an umbrella term for non-mediated food-related actions in people’s daily routines. In their taxonomy, Stok et al. (2018) speak of ‘dietary behavior’, which is subdivided into three areas. Since the term ‘food behavior’ seems to be used more often and more broadly in the scientific articles we reviewed, we decided to use the term ‘food behavior’ instead of ‘dietary behavior’. The term ‘food behavior’ as such can be understood as a ‘set of behaviors’ [28] and can be subdivided into the three areas of *food choice* (preferences, preparation, and intentions), *dietary intake* (healthy or unhealthy, food components, etc.), and *eating behavior* (habits, occasions, portions, etc.) [25]. Analog food behavior encompasses all actions in the three areas of food behavior that may result from combinations of different operating biological, psychological, and socio-cultural variables, and conditions. According to Contento and Koch’s social-ecological model of food behavior [26], food behavior is determined by a conglomerate of different variables and processes operating in three distinct, interrelated domains: the *food-related*, the *person-related,* and the *socio-ecological domains*. The food-related domain includes two dimensions: the biologically defined predispositions (biological dimension) and experience-related variables (experience-related dimension). Biological predispositions (such as taste, hunger, genetics, etc.) and personal experiences with food through physiological (aversions and learned safety) and social conditioning (e.g., parental practices and social networks) determine individual food preferences and aversions and thus, govern the food behavior, especially in the form of taste and affective factors. The person-related domain portrays intrapersonal motivational and facilitative, as well as interpersonal determinants (family practices and social networks), that shape food behavior in the form of beliefs, attitudes, norms, knowledge, and skills. The socio-ecological domain illustrates influences on food behavior that act as variables in the food environment (availability, accessibility, and quality), the socio-cultural environment (e.g., social settings, social attitudes, and cultural practices), the economic environment (e.g., price and time), and the information environment (media and advertising). Thus, in this model, communication is anchored in the information environment and only as media or marketing communication where it is understood as persuasive communication. Other models often used in studies of food behavior also locate communication in this way or do not address it at all because they usually examine individual behavioral variables independent of the social (communication) context in which they may arise or change (e.g., the theory of planned behavior [29], health belief model [30], etc.). Although the socio-ecological model is one of the most comprehensive general models of food behavior and is used as the basis for synthesizing findings in this review, it will be questioned here from the very outset whether the location of the media, especially the newer social media, in the information environment is viable and whether the assumption of an exclusively persuasive, teleological understanding of communication is still contemporary.

To later derive more nuanced conclusions regarding the linkages of digital food communication and analog food behavior, the findings and conclusions of the reviewed studies are systematized according to the variables mapped in the domains of the socio-ecological food behavior model [26], and the three areas of food behavior: food choice, food intake, and eating behavior [25].

## 3. Materials and Methods

The question that guided the scoping review was ‘What is known about the linkages of social media food communication and users’ analog food behavior?

### 3.1. Search Strategy

The Preferred Reporting Items for Systematic Reviews and Meta-Analyses extension for Scoping Reviews (PRISMA-ScR) checklist was used to guide the review process [31]. However, as some items did not fully apply to the approach of this scoping review, some of the individual sections of the PRISMA reporting guidelines were treated as optional and the original designations were not adopted in some cases.

In November 2020, the databases Web of Science and PubMed were searched to identify relevant studies. The search used several combinations of the key terms ‘food behavior’, ‘social media’, and ‘communication’, as well as closely related variations of these three key terms (i.e., ‘eating’, ‘nutrition’, ‘action’, ‘practice’, ‘platform’, ‘Instagram’, ‘Facebook’, ‘talk’, ‘visual’, and ‘post’). Supplemental Table S1 in the Supplementary Material provides the final search strategies upon which the Web of Science and PubMed searches were based. Search terms were customized for each database and combined with Boolean operators to narrow the number of records. We identified 102 records from the Web of Science database and 177 records from the PubMed database. All retrieved records were imported into the Citavi software (Version 6), and duplicate records (*n* = 12) were removed. The remaining 267 records were imported into MaxQDA 2020 and their titles and abstracts were screened for eligibility criteria.

### 3.2. Eligibility Criteria

**Figure 1:**
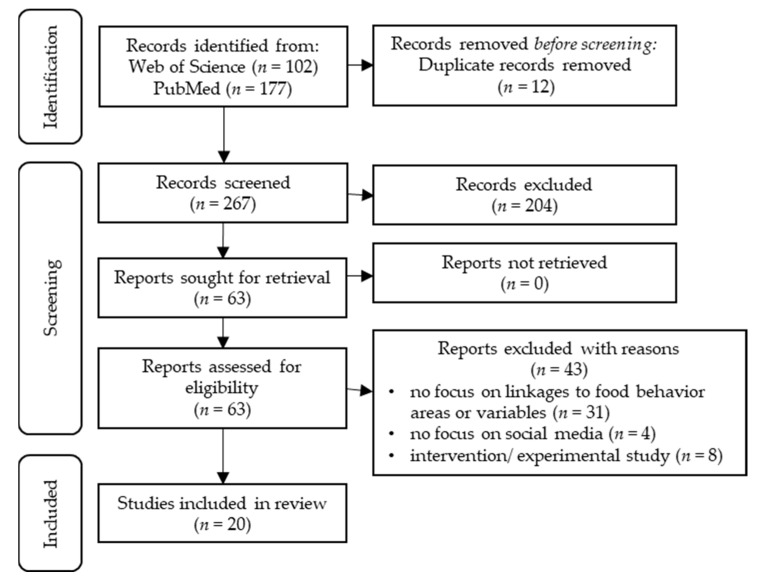
Flow diagram of the article selection process.

To be included in the scoping review, articles had to meet the following criteria: (a) peer-reviewed journal articles; (b) in English; (c) empirical studies or theoretical contributions; (d) published between 2004, when the first popular social media platform Facebook was launched, and November 2020, when the search took place. Qualitative, quantitative, and mixed-methods studies were considered. Articles were excluded if they did not fit into the thematic and conceptual scope of this review. All articles that did not examine or address either digital food communication or analog food behavior were excluded. In the first step of the screening process, 204 records were excluded and 63 were selected for full-text retrieval. The 63 full texts were retrieved and imported into MaxQDA 2020 for a second screening. The full texts were screened in detail to determine whether they establish a link between digital food communication and analog food behavior. Since the particular focus of this review was on the relation between digital food communication and analog food behavior, all studies were excluded that offered no information on this relation, neither in their theoretical preconception nor in the results nor interpretations. In this step, 43 articles were excluded. These articles did not examine or draw conclusions about any area or variable of food behavior (*n* = 31), did not focus on any social media platform (*n* = 4), or reported results of intervention studies or experimental studies with social media as part of an intervention strategy (e.g., for transferring information) and not as the subject of the study (*n* = 8). Finally, 20 full-text articles were included in the analysis that provide evidence or from which conclusions could be drawn on the linkages of digital social media food communication and analog food behavior (see Figure 1). The full list of included articles can be found in Table S2 in the Supplementary Material.

### 3.3. Data Extraction and Analysis

Each of the 20 articles was analyzed in detail in MaxQDA 2020 using deductive and inductive codes as data extraction categories. Standardized category definitions were developed by the authors to guide the analysis. Deductive categories included categories on document details (bibliometric data) and categories derived from the scoping review’s epistemological interest (e.g., scientific discipline, methodology, theoretical reference frames, investigated social media platform and communication mode, understanding of food behavior, relevant results on (or conclusions about) food behavior and determining variables of food behavior, understanding of communication, and conceptual relationships of communication and behavior). The deductive categories were supplemented with inductive categories to further systematize the approaches and findings. To further structure the findings, during the inductive coding process we aligned and subdivided the deductive categories of food behavior and food behavior variables according to the theoretical constructs provided by the areas of food behavior [25] and the social-ecological model of food behavior [26] where appropriate, as described in the second section of this article. Table S3 in the Supplementary Materials provides a structured overview of the coding categories, subcodes, and sub-subcodes of food behavior areas and food behavior variables as they guided the initial analysis. 

## 4. Results

### 4.1. Overview of Included Studies

The exclusion of a further 43 studies in the second screening process illustrates that there are a limited number of studies that focus precisely on the nexus between digital food communication and analog food behavior. Although there are a large number of studies that focus on the nexus between social media and food behavior, there is little scientific literature that conceptualizes or reflects this on a communicative level.

The 20 articles included in this review spanned from 2017 to 2020. Articles published in 18 journals were included, indicating the interdisciplinary interest in the linkages of digital communication and analog food behavior. According to the theoretical reference frames, methodological approaches, and epistemological interests, most articles can be assigned to the sub-disciplines of health communication (*n* = 6) and health psychology (*n* = 2), as well as to the interdisciplinary research field of public health (*n* = 6). The remaining articles were distributed among the research fields of consumer sciences (*n* = 2), digital humanities (*n* = 2), gastronomic sciences (*n* = 1), and sociology (*n* = 1). Of the twenty articles, five addressed digital food communication on the social media platform Instagram, four addressed YouTube, three addressed Twitter, two addressed Facebook, one addressed WeChat, and one addressed the travel social media platform TripAdvisor. One of the articles examined online forums in which users talk about using different social media platforms to manage their everyday food behavior, and three articles did not specify a social media platform and related their statements on the linkages of digital food communication and analog food behavior to social media platforms in general. In keeping with the diversity of social media platforms that were the subject of or referenced in the studies examined, the modes and signs of communication assessed are also diverse. They range from textual to visual (images), to audio-visual (videos), to digital rating signs such as likes, to content indexes such as hashtags, and geographic indexes such as geotags. To the extent that we were able to identify specifics between different modes of communication and evidence or inferences of analog food behavior during our analysis, we describe this in the next section when presenting the results.

### 4.2. Synthesis of Results

The 20 studies examined were all related to food communication and/or behavior, with either one of the two being the subject of the investigation or the subject of knowledge. Differentiating the results according to the three areas of food behavior [25] reveals the diverse aspects of food behavior for which a linkage with digital food communication could be demonstrated. However, it also shows that food behavior is a broad term that can cover a variety of aspects that can be described as food choices, dietary intake, or eating behaviors. Of the 20 studies analyzed, 19 studies produced results on all three areas of food behavior, with proportionately the most results on eating behavior (*n* = 11), then food choice (*n* = 10), and finally dietary intake (*n* = 7), which are described below in this order. The study by Davies et al., [32] which is not included in these 19 studies, conceptualizes the linkages of food communication and food behavior only hypothetically and assumes only abbreviated modelling of the influence of food communication on human perception. Nevertheless, it was included in the reviewed sample because it provides important insights into the influence of social media food communication on food behavior variables.

#### 4.2.1. Linkages of Digital Food Communication and Analog Eating Behavior

**Figure 2:**
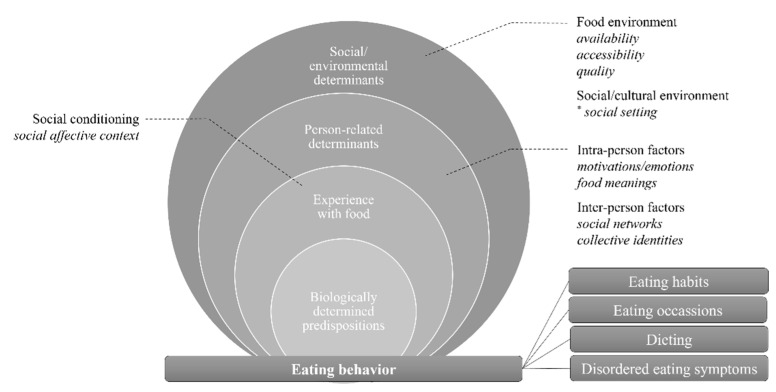
Food behavior variables of analog eating behavior linked to digital food communication (adapted from Contento and Koch, 2021, p. 50). * Variable not directly investigated in reviewed studies, but inferred from results of review.

Eating behavior is the specific area of food behavior that entails behaviors and outcomes related to the actual act of eating [25]. The review found that in the area of eating behavior, habits, occasions, dieting, and disordered eating may be linked to social media food communication. These associations are established via food environment variables, intra- and interpersonal factors, and/or social conditioning factors that are either generated or represented by food communication or directly or indirectly affected by social media food communication (see Figure 2).

In the experience-related dimension, the studies reviewed indicate that social media platforms function as a social affective context that provides a digital platform for the social conditioning of analog eating behaviors. This was particularly demonstrated by Marino (2018) regarding eating habits and mealtime socialization when using digital platforms, which provide virtual spaces for experiencing commensality and reproducing familiar eating routines when eating together with family members and relatives across physical boundaries. In this context, the linkages of social media food communication and analog food behavior are described as performative, in that the use of social media to communicate about and during eating directly influences eating behavior outcomes. In this sense, experiencing virtual commensality and performing typical eating habits manifests itself by ‘doing food due social media’ [33,34]. Although not directly referred to in the studies reviewed, social media usage creates a virtual social setting in which eating happens time-shifted or simultaneous, and social influences and cultural practices are visually or audio-visually represented and indirectly affect the social media user’s analog enacted eating behaviors via cross-cutting variables of the experience- and person-related domain. Since the variable ‘social setting’ was not directly addressed in the reviewed studies but can be abstractly derived from the results of the review, we have marked it with an asterisk in Figure 2. This variable illustrates particularly that social media and media, in general, can no longer be located only in the informative environment but represent a digital space for exchange and interaction concerning food and nutrition.

Within the person-related domain, intra- as well as interpersonal determinants are affected by social media food communication and are inferred to subsequently inform analog eating behaviors. It is argued that eating habits or dieting practices are governed through digitally conveyed food meanings [35,36,37,38] and virtual social relationship networks that serve as collective identity pools that construct and provide symbolic structures for the orientation of analog eating behavior practices [38]. Additionally, motivational factors and emotions are described as variables inspired and triggered by engaging in social media food communication [37,39,40]. However, these are exclusively conclusions regarding the linkages of social media food communication and analog eating behavior, which are mostly theoretical (e.g., based on social identity theory and/or practice theoretical approaches) or hypothetically derived (mostly based on the state of research in a particular field). Equally hypothetical are the studies that view social media food communication as representative of specific analog eating behaviors or related variables. In particular, emotional aspects of eating (especially through symbols such as hashtags) are considered to be reflected through food communication on social media [35]. This goes so far as to consider food communication on social media as a medium of analysis to identify users with symptoms of an eating disorder and to draw conclusions about the likelihood of a disorder from the food concerns expressed in users’ food communication [41].

Variables located in the socio-ecological domain of food behavior are described as influencing analog eating behavior by acting as digitally represented analog material structures. Variables in the food environment (availability, accessibility, and quality) have been found to have a particular impact on analog enacted eating occasions. Thus, providing social media users with an orientation frame to inform the configuration of their analog eating occasions a dependence on the digitally mediated realities regarding the characteristics of the given space and its material possibilities [35]. At the same time, the availability of these food environment variables on social media platforms is considered and used as a data pool to conclude on users’ eating behaviors by deriving quantitative data on the expressions of the variables from social media food communication (especially on eating habits and occasions or disordered eating behaviors (obesity)) [36,42,43,44]. In the latter, however, no linkages are drawn between digital communication and analog behavior, but it is simply assumed that analog eating behavior can be explained and described by digitally represented food environment variables as if the environment variables alone determined the available options for analog eating behavior. Similar approaches have been identified in the area of food choice, where food communication and food information behaviors are viewed as simple reflections of analog food choice behaviors [45]

#### 4.2.2. Linkages of Digital Food Communication and Analog Food Choice

**Figure 3:**
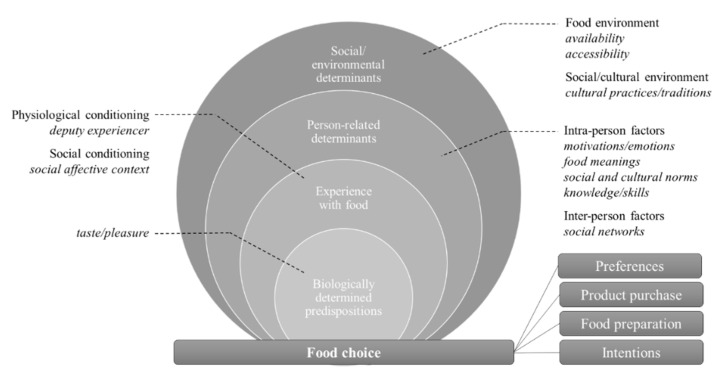
Food behavior variables of analog food choice linked to digital food communication. (adapted from Contento and Koch, 2021, p. 50).

Food choice is the area of food behavior that precedes actual food consumption (e.g., intentions, food preparation, purchasing behavior, and preferences) [25]. The studies reviewed indicate that variables of all domains of the socio-ecological food behavior model are linked to digital food communication in food choices (see Figure 3).

Starting with the variable ‘taste’ as an originally biologically determined predisposition, Onorati and Giardullo (2020) show how taste is re-mediated on TripAdvisor through food communication and conclude that social media users are now socialized in their food behavior in multiple ways, leading to the emergence of new, socially informed preferences and food choice patterns. With this conclusion, the boundaries between the previously biologically determined dimension and the other three dimensions of the model of food behavior become blurred. In the experience-related dimension, personal physiological conditioning becomes less important, as other communicators now share their experiences with certain foods, dishes, or restaurants online, evaluate them, and thus, act as deputy experiencers. These deputy experiencers create meanings for food and a social context of orientation for good taste and its adoption, creating a virtual space for social (re)conditioning [36] that influences analogous food choice behaviors in the form of behavioral intentions and product purchases [46].

Similarly, the variable of taste is coupled with variables of the socio-cultural environment that is reflected, generated, and maintained through digital food communication. For example, cultural practices and traditions are maintained through social media communication and interaction across geographic boundaries and can still determine which foods are considered preferred, even if one leaves the original socio-cultural environment [33]. At the same time, communicating about accumulated food choices and eating experiences, as well as following and networking with vicarious experiencers on social media platforms serve as a means to express adherence to digitally mediated social and cultural norms and form relational networks with like-minded eaters [43], and thus, have a recursive and cross-domain effect on the person-related domain of food behavior variables. Variables such as knowledge or social and cultural norms are elevated from a formerly intrapersonal level to an interpersonal level through food communication on social media, blurring the boundaries of categories even within domains. Online communities serve as culinary support by providing guidance, motivation, and inspiration for using and recombining, contributing to a reservoir of practical knowledge within online communities that guides member users in analog food preparation and planning [33,40,47]. Thus, engaging in social media food communication is understood as a reciprocal process in which different variables of the food behavior model interact and influence each other and affect certain outcomes of food choice behavior.

However, in the sample studied, some studies assume a linear relationship between social media food communication and analog food choice behavior outcomes. In particular, regarding children as a user group, it is assumed that communication about certain foods perceived as unhealthy, e.g., by YouTube influencers, may have a persuasive effect and lead to unfavorable food choices among children [48]. Similarly, in the socio-ecological domain, food environment variables are linked linearly to digital food communication and influence food choice behaviors, such as intentions and product purchase, by serving as transferable information. For example, choosing lunch in an environment with many offerings is made easier by visually documenting the offerings and posting them with geotags on social media platforms. Users are thus informed about variables such as accessibility and availability in a limited geographic area, and the mere availability of the information, it is reasoned, conditions an impulsive imitation of food choice behaviors [35].

#### 4.2.3. Linkages of Digital Food Communication and Analog Dietary Intake

**Figure 4:**
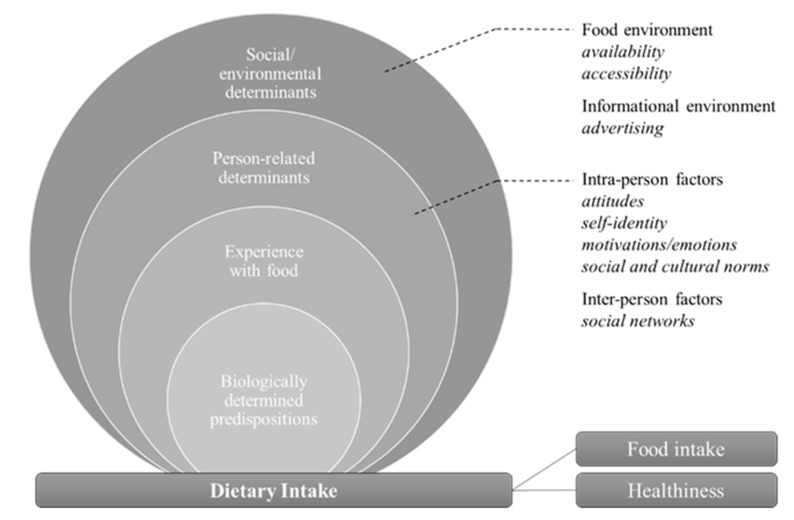
Food behavior variables of analog dietary intake linked to digital food communication. (adapted from Contento and Koch, 2021, p. 50).

Dietary intake is the area of food behavior that includes outcomes related to what is consumed (healthiness, dietary patterns, and food components). This area of food behavior is also referred to as ‘nutrition’ because it directly addresses calorie and nutrient content and the associated characteristics of dietary patterns [25]. In the studies reviewed, the area of dietary intake is the only one for which there is empirical evidence of a direct link between communication and behavior. This may stem from the fact that outcomes in this area, particularly health value and dietary intake, can be measured directly. However, this field assumes a very abbreviated, linear understanding of the linkages of food communication on social media platforms and the analog behaviors performed. The intervening human perception and potentially effective behavioral variables are only perceived as information variables if they are addressed at all. Compared to the areas of food behavior described above, dietary intake also addressed the fewest behavioral variables, with links made only to determinants in the socio-ecological environment and the person-related domain (see Figure 4).

Studies that attempt to explain the linkages between food communication on social media and dietary intake outcomes are typically behavioral science studies that are experimental in design and measure the effects of exposure to food communication on behavioral outcomes (e.g., immediate food intake) in laboratory settings [49]. It is postulated that advertising, as a determinant of the information environment, can directly influence dietary intake. When considering variables of the person-related domain (e.g., attitudes, self-identity, motivations and emotions, or social and cultural norms), it is assumed that these are also influenced by the information environment variables without making them directly the object of study [44,50,51]. Alternatively, the variables of the person-related domain itself, such as social networks, are considered a context in which exposure presupposes adoption of the postulated behaviors, attitudes, etc. [39,42,50]. These hypotheses are derived less from empirical findings than from behavioral theories and models (e.g., social learning theory, impression formation theory, and the state of research in the respective field) in which the respective variables have already been associated with the resulting behavior. However, these models did not establish links between communication and behavioral variables, so the conclusions appear poorly grounded. In studies from the field of digital humanities, food communication itself serves as the object of analysis, using the same approaches and models as in health psychology studies to derive statements regarding person-related determinants as well as the outcomes of dietary intake and the health value of the diet. The textual content of food communications on social media is often combined with indicators of food environment variables (such as geotags, hashtags, or statistical regional data) to describe the dietary intake and health status of entire regions [42,44].

## 5. Discussion

### 5.1. Summary of Evidence

This review aimed to provide a nuanced overview of how the impact of social media food communication on analog food behavior is considered in relevant studies and what is known about these linkages. Findings and evidence on the linkages of digital food communication and analog food behaviors were systematically and critically reviewed and mapped by referring to a more sophisticated distinction of food behaviors into the three behavioral areas of eating behavior, food choice, and dietary intake [25] and attributing the findings identified in the studies to a socio-ecological food behavior model that maps numerous behavioral variables into three domains [26]. By referring to the three areas of food behavior, it was possible to show that research on linkages has so far referred to quite different areas of food behavior without making this explicit. It is, therefore, not possible to speak in general terms of the influence of social media food communication on analog food behavior. Although based on the social-ecological model of food behavior and the food behavior variables depicted, it could be shown that mainly variables of the person-related domain and the social-ecological environment determine the linkages of digital communication and analog behavior, no overarching findings on the three areas of food behavior and the influence of social media food communication on analog behavioral outcomes can be demonstrated. Consequently, it is not possible to summarize comprehensively what statements can finally be derived regarding the impact of social media food communication on the food behavior of users in everyday life.

**Figure 5:**
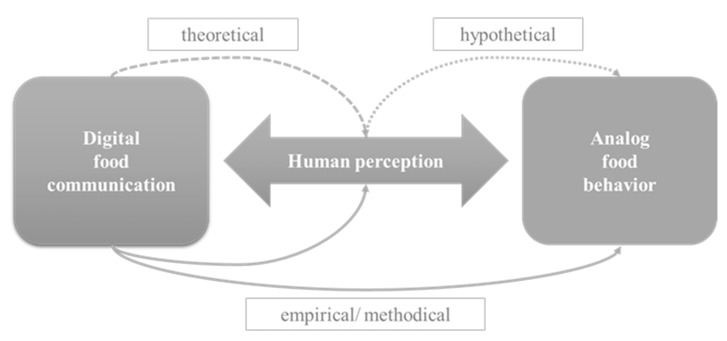
Analytical model of approved linkages of digital food communication, human perception, and analog food behavior.

Furthermore, in eating behavior, we demonstrated how digital and analog behavior become indistinguishable by using social media for food communication, and how food communication has a performative effect on eating behavior through various food behavior variables or enables it at the outset. In the area of food choices, it has also been shown that the links between food communication in social media and analog food behavior are reciprocal, that the boundaries between and within the domains of food behavior variables are blurred, and that behavior variables not only impact food behavior but also exert an impact on each other. Thus, digital food communication on social media platforms becomes more than an additional external variable in the information environment, where it is situated in the behavioral domain of dietary intake. Two central aspects crystallize from the review as fundamental for the fact that linkages between digital food communication and analog food behavior can be established at all: Firstly, the understanding of communication underlying the studies and the links this enables between communication and food behavior variables, and secondly, the fact that it is only through the usage of social media for food communication and the perception of users that linkages of digital communication and analog food behavior become possible. Analytically, this can be summarized in terms of the evidence of the studies reviewed by locating the field of human perception between digital food communication and analog behavior (see Figure 5).

Some studies reviewed provided empirical and methodically derived insights into the linkages between digital food communication and human perception and directly between digital food communication and analog food behavior. At this stage, however, social media food communication primarily represents the medium of analysis from which findings on analog food behavior are derived. No interlocking linkages between digital communication and analog behavior are established. For example, the analysis of users’ food communication revealed that they may suffer from an eating disorder [41], social media analytics of digital humanities showed how people in certain geographical areas eat [42], and analysis revealed their preferences or attitudes towards food [45]. Fewer studies consider analog food behavior as an object of investigation. When they do, they typically test how social media food communication affects food behavior in experimental settings—unidirectional, for example, measured by food intake [49]. In other studies, human perception serves as the object of inquiry, for example, to substantiate the credibility users attribute to digital food communication [39,40,46,52] or the impact of social media food communication on perceived well-being [37,39] or body image [32]. In most cases, however, only hypothetical or no conclusions are then drawn regarding analog food behavior. Similarly, studies that take social media food communication as a starting point and medium of analysis, then theorize inferences regarding human perception and draw hypothetical conclusions about possible food behavior outcomes [33,35,36,38]. Thus, the evidence provided by the studies reviewed regarding the relationship between digital food communication and analog food behavior is insufficient. That is unless one understands analog food behavior to be only food intake and assumes that the experimental exposure to social media food communication and the information provided by the study participants regarding their social media use are sufficient, in order to demonstrate an actual link between communication and behavior. However, this would contrast with the studies examined, which first theoretically derive the links between food communication and human perception and then hypothesize what specific variable features of human perception might mean for specific analogous outcomes of food behavior (including eating behavior and food choices).

### 5.2. Interpretation

The basis on which the linkages of digital communication and analog behavior are justified depends on whether a direct link between communication and behavior is described, whether human perception is still considered between digital communication and analog behavior, and whether the influence of communication on human perception is also investigated in the respective studies or even takes center stage. Accordingly, some studies provide evidence of the links between digital communication and human perception and conclude only about the links between human perception influenced through digital food communication and analog food behavior. The analysis of the studies revealed that how the studies substantiate the linkages of communication and behavior is dependent on the understanding of communication and thus, on the theoretical reference frames on which the studies are based. These understandings of communication were not explicitly stated in any of the studies, and no study explicitly referred to the relevant communication theories, but the authors of this review reconstructed the understandings by looking more closely at the studies’ theoretical frames of reference. Most studies take a linear understanding of communication as a basis (*n* = 8), with seven studies assuming a persuasive influence of communication on behavior and one study modelling communication as merely conveying information. Another seven studies in this review view digital food communication as representative of analog food behavior or its determinants. Two studies assume a reciprocal understanding of communication, and three studies understand the linkages between communication and behavior as performative. Those studies that assume a reciprocal and performative understanding of communication explain the linkages of digital food communication and human perception theoretically or methodologically, and the linkages of human perceptual variables, altered by communication, and analog food behavior outcomes hypothetically. Although they do not provide empirical evidence of specific food behavior outcomes that are influenced by food communication, their conclusions are compelling, especially given the theoretical references and underlying constructivist understanding of communication.

These insightful studies assume that the symbolic and socio-cultural variables of the domains that determine food behavior are generated, reproduced, and reflected through communicative processes [53,54]. Digital food communication is thus not only a means of conveying information with possible persuasive effects but rather a virtual communication and interaction space in which—unlike in analog contexts—food-relevant socio-cultural structures are produced and reproduced, which can act as variable expressions of food behavior [55,56]. This social science-based argument underscores the assumption of the duality of structure and action—that both individual and socio-cultural factors must be considered to understand and explain human behavior [57]. People act in certain ways and certain situations, but their behavior is determined by factors that lie outside the situation and are part of the socio-ecological context. Social media platforms represent such contexts in which socio-ecological variables and in particular socio-cultural structures are constantly and fluidly (re)produced. These structures frame individual analogous behavior, in that individuals who use social media to inform themselves or communicate about food agree over time through interactions on a shared definition (frame) of a situation in which they can act meaningfully and adapt their behavior to their own and other participants’ expectations [58]. However, these frames do not completely determine individual analog behavior. Rather, the structural aspects of social reality are (re)produced by individual action digitally and analogously, on- and offline. In this sense, individual analog food behavior and digitally re(produced) socio-ecological structures are linked by the two processes of framing and re(production); both processes are enabled and mediated by communication [59]. For the social structures constructed and mediated in digital food communication to become meaningful and be reproduced by users in their everyday lives, users must perceive them and ascribe meaning to them [60]. To use network technology terminology, digital food communication becomes significant in analog lives when the digital information is modulated into meaningful analog signals for the users. In this sense, the explanatory variables of food behavior in the different domains of the socio-ecological model of food behavior [26] could act as modems to which digital information can dock and be converted into analog signals. Therefore, this perspective draws attention to how individuals perceive and make use or sense of social media food communication so that it affects their analog food behavior.

### 5.3. Implications for Research and Practice

In describing and explaining the linkages of digital food communication and analog food behavior, none of the studies reviewed relied explicitly on food behavior models. The same applies to the description of the linkages between food communication and human perception. Only by referring the study results back to the socio-ecological food behavior model and the three areas of food behavior, was it possible to differentiate and relate them to the subject of the matter. Traditional approaches to food behavior no longer capture the complexity of food actions in digitalized societies and require an update with the emergence and increasing usage of social media [27]. Behavioral models that understand communication as an external variable with persuasive, linear influences on behavior are no longer valid in today’s mediatized food world. Therefore, there is a great need to test and further develop food behavior models for their explanatory power for the food behavior of media users and especially social media users. Nowadays, people use and interact with social media, and there is no unidirectional influence, but many intermediary co-constructs that are seemingly unrecognized and unexplained so far but are relevant for food behavior.

Our findings highlight that the theoretical frame of reference for communication and its function in studies investigating the relationship between digital food communication and (analog) food behavior need to be more clearly defined and elaborated. Defining the function of communication and its relation to behavioral variables is an important condition for studying the relationship between social media and analog food behavior. Examining and understanding the linkages of digital food communication and analog food behavior in more detail offers an opportunity to advance the consideration of communication and media in current theorizing and modelling of food behavior, and to complement previous approaches with a more contemporary perspective on modern social communication forms and modes. Moreover, a critical analysis should not only examine how food communication on social media platforms affects food intake or individual variables of dietary behavior, but also consider the underlying mechanisms that lead people to accept and adopt digitally constructed and mediated norms and values (e.g., through alignment with their self-perceived identity, social relationships, the language of food-related experiences, and spatio-temporal environments, etc.). Social media as contemporary communicative social environments require researchers to enter new areas of theoretical and methodological consideration and to engage in interdisciplinary and networked approaches to the objects of digital food communication, human perception, and the outcomes of analog food behavior in the research process [61]. Only by considering all three areas in the research process, and with a sophisticated understanding of communication appropriate to the use and perception of such digital spaces, can valid findings be generated and meaningful conclusions derived regarding the connections between digital food communication and analog food behavior.

For practitioners, the findings of the review and their discussion also provoke reflection on the relevance and potential impact of food communication and public health experts on social media platforms. The linkages of digital food communication and analog food behavior have not yet been fully understood, but the review has found that social media and digital food communication enable new ways of constructing, expressing, and influencing food behavior variables that can be transformed into analog food behavior outcomes through complex mechanisms. To conclude that there is a unidirectional relationship between the sharing of food information on social media and the promotion of desirable analog food behaviors would, therefore, be very ambitious. Nevertheless, it is important and right that nutrition and health professionals also position themselves and show their presence on social media. However, to date, research has generated insufficient evidence on compatible communication strategies and different types of social media users and usage practices to assess the relevance of additional information dissemination via this channel for analog outcomes in food behavior.

### 5.4. Strengths and Limitations

The strengths of the review consist of the interdisciplinary compilation of the analyzed studies. Thus, we were able to integrate study designs with different and diverse theoretical foundations and identify a wide variety of approaches and previously found linkages between social media food communication and analog food behavior. At the same time, this selection process can be seen as a limitation of the review, as it is not entirely clear why, for example, studies that nevertheless measured certain outcomes of dietary intake were included. This is because we focused our selection on studies that explicitly addressed food communication or referred to synonyms or terms with similar meanings. This may have excluded studies that demonstrate or address relationships between social media, food behavior variables, and food behavior outcomes but do not address communicative aspects. If communicative aspects are not addressed at all in the investigation of the influence of social media on food behavior, then this also seems surprising since social media is mainly constituted by communication and interaction. However, this review through the novel analytical approach and focus provides the first comprehensive assessment and contextualization of the underlying implicit assumptions regarding the potential impact of digital communication on analog food behavior on which the claims regarding the seemingly vast influence of social media on our food behavior are based.

One limitation, however, is that the data collection took place at the end of 2020 and the analysis was based on articles that had been published by that time. We did not include more recent studies in the analysis and the results section, but when finalizing the article in 2022, we revisited recent studies and compared their approaches and results regarding the linkages between digital food communication and analog food behavior. No correlations were discovered that had not already been illustrated in our review.

A further limitation is that the groups of social media users and research participants addressed in the studies were not specified in the review. The sample sizes, age groups, and locations of the studies are listed in Table S2 in the Supplementary Material. These data do not help to better explain the identified relations between digital food communication and analog food behavior. However, they do show that the majority of empirical research is on populations in the Global North and mostly focuses on children, adolescents, and young adults. They also point to the heterogeneity of potential data that can be used to analyze the context of interest. Most often, either textual or visual data available online are analyzed, or empirical studies are conducted with users of social media platforms; rarely are these two types of data combined in the research process.

A further limitation is that the correlations between the individual model variables of food behavior moderated by digital food communication were presented as examples and were not described holistically. Consequently, it is not possible to derive precise conclusions about the mechanisms of the relationship between digital food communication, human perception, and analog food behavior. Similarly, the various modes of communication examined in the studies were addressed only by way of example, insofar as a particular link or significance was emphasized or suspected. However, since the purpose of this review was to investigate the relationships between digital food communication and analog food behavior, the reference to previous interdisciplinary findings on the systematic differentiation of food behavior domains and potentially effective food behavior variables can be seen as a major strength of the review. Furthermore, the review can be seen as a groundwork for future research on contemporary food communication and behavior models and as a wake-up call for food and nutrition research to systematically approach this research area and to further advance and update existing models and theories of food behavior.

## Supplementary Materials

The following supporting information can be downloaded at: https://www.mdpi.com/article/10.3390/ijerph19158990/s1. Table S1 in the Supplementary Material provides the final search strategies on which the Web of Science and PubMed searches were based. Table S2 in the Supplementary Material shows the full list of included articles. Table S3 in the Supplementary Materials provides a structured overview of the coding categories, subcodes, and sub-subcodes of food behavior areas and food behavior variables as they guided the initial analysis.
