# Supplementary material for: Exploring the Linkages of Digital Food Communication and Analog Food Behavior: A Scoping Review
Source: Int J Environ Res Public Health. 2022 Jul 24;19(15):8990. doi: 10.3390/ijerph19158990 (PMC9332013; doi:10.3390/ijerph19158990)
Supplement: Supplementary file 1 [file ijerph-19-08990-s001.zip › ijerph-1761844-supplementary.pdf]

International Journal of
Environmental Research and Public Health

MDPI

Supplementary Material

Table S1: Database search strings.

Database	Search string
Web of Science	((TS=(food AND behavior OR food AND behaviour OR food AND action OR food AND practice OR food AND practices OR food and beliefs OR food AND thoughts OR nutrition AND behavior OR nutrition AND behaviour OR nutrition AND action OR nutrition AND practice OR nutrition AND practices OR nutrition and beliefs OR nutrition AND thoughts OR eating AND behavior OR eating AND behaviour OR eating AND action OR eating AND practice OR eating AND practices OR eating and beliefs OR eating AND thoughts) AND TS=(social AND media OR social AND media AND platform OR instagram OR facebook OR pinterest OR twitter OR youtube OR tiktok OR tik AND tok OR online O R internet) AND TS=(communication OR tweets OR post OR posts OR pics OR pictures OR talk OR pins OR video OR videos OR story OR stories OR visual OR sharing OR share OR behavior AND change OR behaviour AND change OR behavior AND Changing OR behavi our AND changing) AND AB=(food AND behavior OR food AND behaviour OR food AND action OR food AND practice OR food AND practices OR food and beliefs OR food AND thoughts OR nutrition AND behavior OR nutrition AND behaviour OR nutrition AND actio n OR nutrition AND practice OR nutrition AND practices OR nutrition and beliefs OR nutri tion AND thoughts) AND AB=(social AND media OR social AND media AND platform OR instagram OR facebook OR pinterest OR twitter OR youtube OR tiktok OR tik AND tok O R online OR internet) AND TI=(food OR nutrition) AND TI=(social AND media OR social AND media AND platform OR instagram OR facebook OR pinterest OR twitter OR youtube OR tiktok OR tik AND tok OR online OR internet))) AND LANGUAGE: (English) AND DOCUMENT TYPES: (Article) Indexes=SCI-EXPANDED, SSCI, A&HCI, ESCI Timespan=2004–2020
PubMed	((((((("nutrition"[Title/Abstract] OR "food"[Title/Abstract] OR „eating"[Title/Abstract]) AND ("behavior"[Title/Abstract] OR "behaviour"[Title/Abstract] OR "action"[Title/Abstract] OR "practice"[Title/Abstract] OR "practices"[Title/Abstract]) OR "beliefs"[Title/Abstract] OR "belief"[Title/Abstract] OR "thoughts"[Title/Abstract]) AND ("social media"[Title/Abstract] OR "social media platform"[Title/Abstract] OR "instagram"[Title/Abstract] OR "facebook"[Title/Abstract] OR "pinterest" OR "twitter"[Title/Abstract] OR "youtube"[Title/Abstract] OR "tiktok"[Title/Abstract] OR "tik tok"[Title/Abstract])("nutrition"[Title/Abstract] OR "food"[Title/Abstract]) AND ("behavior"[Title/Abstract] OR "behaviour"[Title/Abstract] OR "action"[Title/Abstract] OR "practice"[Title/Abstract] OR "practices"[Title/Abstract]) OR "beliefs"[Title/Abstract] OR "belief"[Title/Abstract] OR "thoughts"[Title/Abstract]) AND ("social media"[Title/Abstract] OR "social media platform"[Title/Abstract] OR "instagram"[Title/Abstract] OR "facebook"[Title/Abstract] OR "pinterest" OR "twitter"[Title/Abstract] OR "youtube"[Title/Abstract] OR "tiktok"[Title/Abstract] OR "tik tok"[Title/Abstract]) AND ("communication"[Title/Abstract] OR "tweets"[Title/Abstract] OR "post"[Title/Abstract] OR OR "posts"[Title/Abstract] OR "pics"[Title/Abstract] OR "pictures"[Title/Abstract] OR "talk"[Title/Abstract] OR "pin"[Title/Abstract] OR "pins"[Title/Abstract] OR "visual"[Title/Abstract] OR "story"[Title/Abstract] OR "share"[Title/Abstract] OR "sharing"[Title/Abstract])) NOT (suicid)) NOT (cigars)) NOT (nicotine)) NOT (vaccine)) NOT (malaria)) NOT (transplantation) Filters: Abstract, Full text, Journal Article, Humans, English, German

Table S2: Overview of studies reviewed.

**Authors**	**Year**	**Journal**	**Sample sizes**	**Age groups**	**location/ language**
Hoffman et al. [52]	2017	Health Communication	N = 637	not specified/ university students	US
Borah & Xiao [46]	2018	Journal of Health Communication	n1 = 340; n2 = 552	M1 = 19.8 years; M2 = 19.1 years	US
Marino [33]	2018	Journal of Material Culture	n = 300 forum posts	not specified	Italians living in the UK
Middha [35]	2018	Communication Research and Practice	n = 16; n = 400 pictures and posts	not specified/ university students	Australia
Easton et al. [39]	2018	Journal of Medical Internet research	n = 20	18–25 years	UK
Raggatt et al. [37]	2018	BMC Public Health	n = 180	M = 23 years	Australia
Cavazos-Rehg et al. [41]	2019	PloS one	n = 2.584 Twitter tweets	not specified	English language
Coates et al. [51]	2019	Frontiers in Psychology	not specified; YouTube Videos	not specified	UK
Coates et al. [49]	2019	Pediatrics	n = 176	9–11 years	UK
Holmberg et al. [40]	2019	Health Informatics Journal	n = 20	13–16 years	Sweden
Huang et al. [42]	2019	International Journal of Environmental Research and Public Health	n = 4.785.104 Twitter tweets	not specified	US
Peng [43]	2019	Social Identities	n = 16	22–27 years	China
Castello-Martinez & Tur-Vines [48]	2020	Clinical Obesity	n = 304 YouTube videos	not specified	Spanish language
Cavazza et al. [50]	2020	Appetite	n = 195	17–60 years; M = 28.12 years	Italy
Coates et al. [47]	2020	International Journal of Environmental Research and Public Health	n = 24	10–11 years	UK
Davies et al. [32]	2020	Body Image	n = 154	18–25 years; M = 21.58 years	UK
Laguna et al. [45]	2020	Food Quality and Preference	n = 362; n = 37.200 English Twitter tweets; n = 14.537 Spanish Twitter tweets	20–76 years	Spain
Walsh & Baker [38]	2020	Food Culture & Society	n = 144 Instagram posts	not specified	not specified
Onorati & Giardullo [36]	2020	Food Culture & Society	n = 2.213 Trip Advisor reviews	not specified	Italy
Vydiswaran et al. [44]	2020	Journal of the American Medical Informatics Association	n = 1.273 Twitter tweets	not specified	US

Table S3: Deductive codes of food behavior areas and variables.

Category	Subcodes	Sub-subcodes
Food behavior (Stok et al., 2018) [25]	Food choice	Preferences<br>Share of income spent on food<br>Willingness-to-pay<br>Frequency of purchase<br>Product purchase<br>Food preparation<br>Intentions
	Dietary intake	Dietary pattern:<br>Type of pattern<br>Diversity of pattern<br>Healthiness<br>Meal pattern:<br>Meal content<br>Caloric and nutrient intake distribution<br>Food intake<br>Food components:<br>Nutrients<br>Energy<br>others
	Eating behavior	Eating habits<br>Eating occasions:<br>frequency<br>time<br>Portions:<br>size<br>number<br>Dieting<br>Disordered eating symptoms<br>Neophobia/ pickiness/ fussiness
Food behavior variables (Contento & Koch, 2021) [26]	Food related domain	
	Biologically determined predispositions	Taste/ pleasure<br>Hunger/fullness mechanisms<br>Sweet, sour, salt, bitter, umami<br>Sensory specific satiety<br>Genetics
	Experience-related Variables	Associative conditioning<br>Physiological conditioning:<br>Familiarity; learned safety<br>Conditioned food preferences<br>Conditioned satiety<br>Social conditioning:<br>Social affective context<br>Parenting practices and styles
	Person-related domain	
	Intra-person factors	Motivational:<br>Beliefs<br>Attitudes<br>Motivations<br>Food meanings<br>Values<br>Cultural and social norms<br>Self-identity<br>Facilitating:<br>knowledge/ skills<br>self-efficacy and self-direction skills
	Inter-person factors	Family practices and social networks
	Socio-ecological domain	
	Food environment	Food availability<br>Food accessibility<br>Food quality
	Social/ cultural environment	Social settings

		Cultural practices/ traditions<br>Social structures/ policy
	Economic environment	Resources<br>Prices<br>Time
	Informational environment	Media<br>Advertising
